# Optimizing subject retention in a longitudinal birth cohort study: lessons learned from the Vancouver site of the CHILD Study

**DOI:** 10.1186/1710-1492-10-S1-A20

**Published:** 2014-03-03

**Authors:** Linda Warner, Mary Ann Mauro, Susan Menzies, Ghazal Assadian, Robby Mamonluk, Claire Lepine, Stuart E Turvey

**Affiliations:** 1 Department of Pediatrics, University of British Columbia, Vancouver, BC, V6T 1Z4, Canada

## Background

The Canadian Healthy Infant Longitudinal Development (CHILD) study is a multicentre birth cohort study following children for the first five years of life to determine how environmental and genetic variables impact early life health, particularly the development of asthma and allergies. Optimal subject retention is essential for scientific integrity, budget containment and ultimately, for continuity of data collection.

We completed an analysis of participants from the Vancouver general cohort after their first year in the study to address retention challenges related to urban mobility, time constraints, and stressful life situations while considering their socio-economic status (SES) in regards to family income and parents’ education level.

## Methods

Reasons for voluntary participant withdrawals were identified by questionnaires and by direct participant feedback. Anonymous surveys were administered to parents to evaluate clinical practice, and provide insight on changes that could be implemented.

## Results

Out of 706 participants the CHILD study successfully retained 93% of participants with 3% excluded at birth due to exclusion criteria (e.g. premature birth, significant medical complication) and another 4 % of the cohort voluntarily withdrawing. 11% of active participants were identified as participants at risk of withdrawing. Issues putting these participants at risk included: lack of time (25%), difficulty with testing (23%) and inconvenience of travel (24%) with a remaining 28% divided into smaller categories affecting the participants such as divorce or health concerns.

The administered anonymous surveys indicated that staff professionalism and ability to establish good rapport, while expressing value and appreciation, were the most important characteristics of the staff to parent participants. The confidentiality of the survey provided an honest outlet for parents to empower them in giving direct feedback, thus improving staff availability and ease of clinic process and procedures. Scheduling flexibility, the use of birthday cards and other monetary reimbursements, as well as the dissemination of knowledge and test results were noted by parents as positive methods that increased participation and retention rates.

## Conclusion

Successfully engaging and retaining study participants are crucial to achieving study objectives and collection of quality of data. Recruitment and retention obstacles should be identified at the onset of a longitudinal study. Adapting to these challenges requires implementation of new strategies and a flexible approach. Continuity of staff service, as well as participant involvement, enhances both the quality of data and the value participants place on study.

